# Sulfur inhibits the growth of androgen-independent prostate cancer *in vivo*

**DOI:** 10.3892/ol.2014.2700

**Published:** 2014-11-11

**Authors:** FEI DUAN, YUHUA LI, LIANGKANG CHEN, XIAOYU ZHOU, JIANXING CHEN, HAILIN CHEN, RUNSHENG LI

**Affiliations:** 1 Shanghai Medical College, Fudan University, Shanghai 200032, P.R. China; 2 Key Laboratory of Contraceptive Drugs and Devices of NPFPC, Shanghai Institute of Planned Parenthood Research, Shanghai 200032, P.R. China; 3 Department of Pathophysiology, Prostate Diseases Prevention and Treatment Research Center, Norman Bethune College of Medicine, Jilin University, Jilin 130021, P.R. China

**Keywords:** sulfur, 22Rv1 cell line, DU-145 cell line, prostate cancer

## Abstract

Sulfur is a bright yellow crystalline solid at room temperature. The aim of the present study was to investigate the inhibitory effect of sulfur on prostate cancer (PCa) *in vivo*. Prostate tumors were developed by injecting 22Rv1 or DU-145 PCa cells into sulfur-treated or untreated nude mice. The weight and volume of the tumors were measured. The cancer cells were separated from the tumors, and analyzed for their growth rate and clonogenicity in culture. The expression of PCa-targeted genes was also assessed using real-time polymerase chain reaction. The rate of growth of 22Rv1 tumors in sulfur-treated nude mice gradually decreased, and was reduced by 41.99% (P<0.01) after 22 days when compared with that of the control group. In addition, the growth of DU-145 tumors was also suppressed by 75.16% (P<0.05) after 11 weeks. The clonogenicity of the sulfur-treated tumor cells decreased by 36.7% when compared with that of the control cells. However, no significant difference in cell growth was identified. mRNA levels of the androgen-receptor, prostate specific antigen and human Hox (NKX3.1) genes were significantly decreased by 32.8, 48.2 and 42.2% in sulfur-treated tumors, respectively. Additionally, it was found that the hydrogen sulfide concentration in the serum of sulfur-treated mice was increased by 4.73% (P<0.05). Sulfur significantly suppressed the growth of PCa *in vivo*. Since sulfur is a known ingredient used in traditional Chinese medicine, it may be used clinically for the treatment of PCa, independently or in combination with other medicine.

## Introduction

Sulfur is bright yellow crystalline solid or powder material. In traditional Chinese medicine (TCM), sulfur is widely used for detoxifying the body and the treatment of scabies (1,2), healing sores and itching (3). It was confirmed by United States Pharmacopeia that sublimed sulfur and subsided sulfur may be used as drugs (4). When absorbed by the skin, sulfur is metabolized to inorganic sulfide or organic sulfocompounds, and it is involved in metabolism *in vivo* (5). Recently, studies have reported that sulfocompounds inhibit cancer cell growth. For example, GYY4137, a sulfocompound, caused the concentration-dependent killing of various cancer cell lines (6), and S-propargyl-cysteine exhibited anticancer effects on gastric cancer cells (7). Furthermore, Allicin, which contains diallyl disulfide, reduced the risk of a variety of malignant tumors, including different glioblastoma cells (8) and hepatocellular carcinoma (9) *in vitro,* and osteosarcoma cells *in vitro* and *in vivo* (10,11).

Prostate cancer (PCa) is the most common malignancy of the male genitourinary system. Since Huggins first treated PCa by androgen deprivation treatment (ADT) (12), ADT has been widely used for PCa treatment. Initially, PCa responds to androgen ablation and disease progression slows down. However, the androgen ablation is palliative, and the disease eventually ensues (13). PCa recurs and the surviving cancer cells become androgen-independent. At this stage, tumors are more aggressive and usually fatal, and hormone blockade therapy fails (14). At present, no effective therapy method has been identified for recurrent PCa and only traditional treatments are primarily used to provide symptomatic benefits, and identifying new methods to treat androgen-independent PCa has been a new area of focus for researchers (15). Recently, the pathways meditated by an androgen receptor (AR) or to bypass ARs have been researched in recurrent PCa, additionally treatment pathways and novel therapies have been investigated to PCa therapy with this potential target Recently, the pathways meditated by an androgen receptor (AR) or to bypass ARs have been researched in recurrent PCa (16,17). Additionally treatment pathways and novel therapies have been investigated for PCa therapy with this potential target (18,19). 22Rv1 is an androgen-independent PCa epithelial cell line, which grows independently of androgen, and is representative of clinical recurrent PCa. However, 22Rv1 cells express the AR and react with androgens (20). Previous studies have shown that the progression of androgen-independent PCa is independent of the androgen, but dependent on the AR signaling pathway (21). 22Rv1 cells also secrete prostate-specific antigen (PSA) (22), which is often used to evaluate the efficiency of PCa treatment. DU-145 and PC-3 cell lines are also androgen-independent PCa cell lines and, thus, are often used in the study of PCa. The two cell lines do not express AR and PSA (23), while the majority of clinical PCa cases significantly express the two genes.

In the present study, 22Rv1 and DU-145 prostate tumors were develoepd in nude mice and the aim of the study was to investigate the inhibitory effect of sulfur on prostate cancer cells.

## Materials and methods

### Drugs and animals

Sulfur powder with a purity of ≥99% was purchased from the Shanghai Chemical Reagent supply station and mixed with milk powder (Nestlé S.A., Vevey, Switzerland) at 1:30 (w/w).

Specific pathogen-free (SPF) male BALB/c nude mice aged between six and eight weeks (weight range, 18–25 g) were purchased from Shanghai SLAC Laboratory Animal Co., Ltd., [License Number, SCXK (Shanghai) 2007–0002; Shanghai, China)], and fed under SPF conditions. Mice in the 22Rv1 and DU-145 experiments were randomly divided into two groups, control and sulfur-treated groups, with 10 mice in each group. Ethical approval for the study was obtained from the Animal Ethics committee of Shanghai Institute of Planned Parenthood Research (Shanghai, China).

### Cell culture

22Rv1 cells were purchased from Shanghai Institute of Cell Life Sciences Resource Center (Shanghai, China). The cells were maintained in RPMI-1640 medium (Hyclone; Thermo Fisher Scientific, Rockford, IL, USA) containing 50 ml/l fetal bovine serum (FBS; Gibco-BRL, Carlsbad, CA, USA), 100,000 units/l penicillin and 100 mg/l streptomycin, at 37°C with an atmosphere of 5% CO_2_.

### Xenograft tumor development in nude mice

22Rv1 cells and DU-145 cells were harvested at the exponential growth stage, washed and suspended in phosphate-buffered saline (PBS). The cells were counted, then the cell suspension was subcutaneously injected into the flanks of mice with 2×10^6^ cells in 0.1 ml PBS. A Trypan-blue exclusion assay was performed to ensure cell viability (>99%) prior to inoculation.

Each mouse in the sulfur-treated group was provided with 0.62 g/day sulfur-milk powder one day following inoculation with 22Rv1 and DU-145 cells, while mice in the control group were provided with milk powder. Tumor size was measured in two diameters every other day (22Rv1 tumor) or every week (DU-145 tumor). Tumor volume (cm^3^) was calculated using the following formula: Tumor volume = a^*^b^2^/2 (a, longer diameter; b, shorter diameter).

Following the experiment, mice with 22Rv1 tumors were narcotized with 0.2 ml 1% sodium pentobarbital and blood was then collected from the heart. Tumors were dissected and weighed.

### Cells separation from 22Rv1 tumors

22Rv1 tumors were cut into sections between 1 and 2 mm^3^, and tumor sections in the same group were mixed, washed with cold PBS containing 200 units/ml penicillin and 200 mg/l streptomycin, digested with trypsin for 5 min at 37°C, and then the digested cells were suspended by RPMI-1640 medium with 5% FBS. The cell suspension was filtrated using a 200-mesh sieve, and then centrifuged at 175 × g for 5 min. The supernatant was then discarded and the cells were suspended with fresh medium, and incubated at 37°C in an atmosphere of 5% CO_2_.

### Clone-forming capability of tumor cells

A total of 300 tumor cells from the sulfur-treated and control groups were seeded, respectively, into 24-well plates in RPMI-1640 medium and incubated for two weeks. The medium was then discarded, cell clones were washed three times with PBS, fixed with 5% formaldehyde for 20 min, stained with crystal violet solution for 5 min, washed for several times, dried and counted.

The cell growth of the tumor cells was measured using the MTT method demonstrated by Meletiadis *et al* (21).

### Analysis of gene expression by quantitative polymerase chain reaction (qPCR)

Total RNA was extracted using TRIzol reagent (Invitrogen Life Technologies, Inc., Carlsbad, CA, USA) according to the manufacturer’s instructions. Total RNAs in the same group were mixed in the same quantity. First-strand cDNA was synthesized from 2 μg of RNA mixture using Quant Reverse Transcriptase (Toyobo Co., Ltd., Osaka, Japan). Gene expression in 22Rv1 tumors was measured using qPCR. The sequences of the primers used are shown in Table I.

### Hydrogen sulfide (H_2_S) levels in mice serum

Blood was collected from the hearts of the mice and the serum was separated. H_2_S levels in the serum were analyzed using the mouse serum H_2_S Elisa kit according to the manufacturer’s instructions (Shanghai Feng Xiang Biological Technology Co., Ltd., Shanghai, China).

### Statistical analysis

Data were analyzed using SPSS software, version 11.5 (SPSS, Inc., Chicago, IL, USA). The continuous variables of tumor weight, tumor volume and H_2_S level were compared using one-way analysis of variance. Consequently, the analyzed results are presented as the mean ± standard deviation (SD). In addition, the clones were also presented as the mean ± SD. P<0.05 was considered to indicate a statistically significant difference.

## Results

### Sulfur inhibits the growth of 22Rv1 and DU-145 tumors

**Figure 1:**
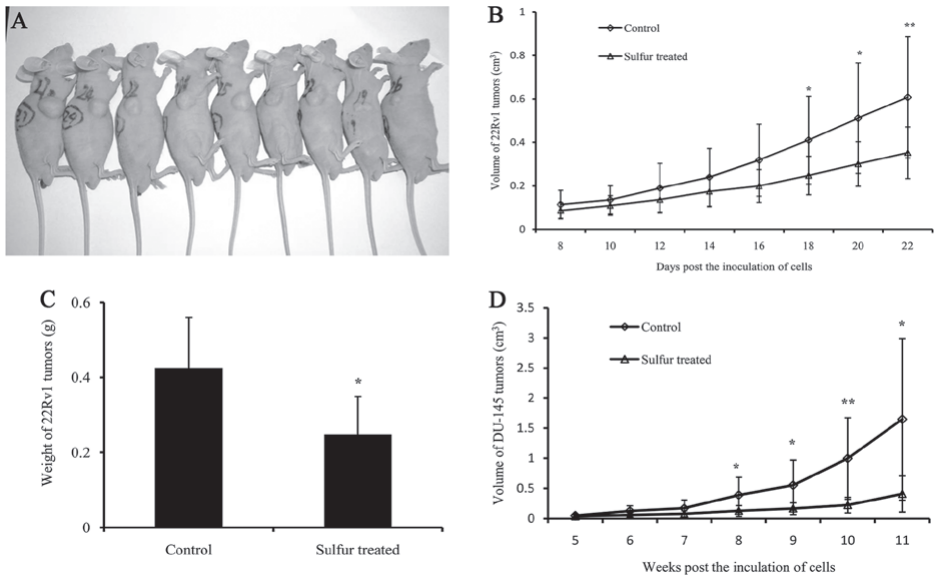
Sulfur inhibits the growth of PCa tumors. (A) A total of 2×10^6^ 22Rv1 or DU-145 cells were inoculated in the flank of each nude mouse. Tumor volumes were measured at the indicated times. (B) Growth of 22Rv1 tumors gradually slowed and showed a significant decrease 18 days following inoculation (C) 22Rv1 tumor weights were measured 22 days following inoculation. (D) Growth of DU-145 tumors significantly decreased after eight weeks following inoculation. ^*^P<0.05 and ^**^P<0.01.

Approximately eight days following inoculation of the 22Rv1cells, the tumor sizes were measurable (Fig. 1A). The growth of the tumors obtained from sulfur-treated mice gradually slowed down, and a significant difference was identified 18 days following inoculation when compared with that of the control group. The mean tumor volume of the sulfur-treated group was 0.35±0.12 cm^3^ after 22 days, which was 41.99% smaller than that of the control group (0.61±0.28 cm^3^) (P<0.01; Fig. 1B). The mean tumor weight of the sulfur-treated group was 0.25±0.10 g, which was significantly decreased by 41.78% when compared with that of the control group (0.43±0.13 g) (P<0.05; Fig. 1C). The mean body weights of the tumor-bearing mice were 21.11±1.62 and 24.91±1.56 g in the sulfur-treated and control groups, respectively. However, no significant difference in body weight was identified. The results demonstrated that sulfur significantly inhibits the growth of 22Rv1 tumors *in vivo*.

DU-145 tumors were measurable five weeks following inoculation, and the growth of tumors of the sulfur-treated group gradually slowed down and a significant difference was identified eight weeks after inoculation, when compared with that of the control group. The mean tumor volume of the sulfur-treated group was 0.41±0.30 cm^3^ 11 weeks after inoculation, which was 75.16% smaller than that of the control group (1.64±1.34 cm^3^) (P<0.05; Fig. 1D). The results demonstrated that sulfur significantly inhibits androgen-independent prostate tumor growth.

Since 22Rv1 cells, but not DU-145 cells, express AR and PSA, 22Rv1 tumors were selected for further study.

### Sulfur decreases the clonogenicity of 22Rv1 tumor cells

**Figure 2:**
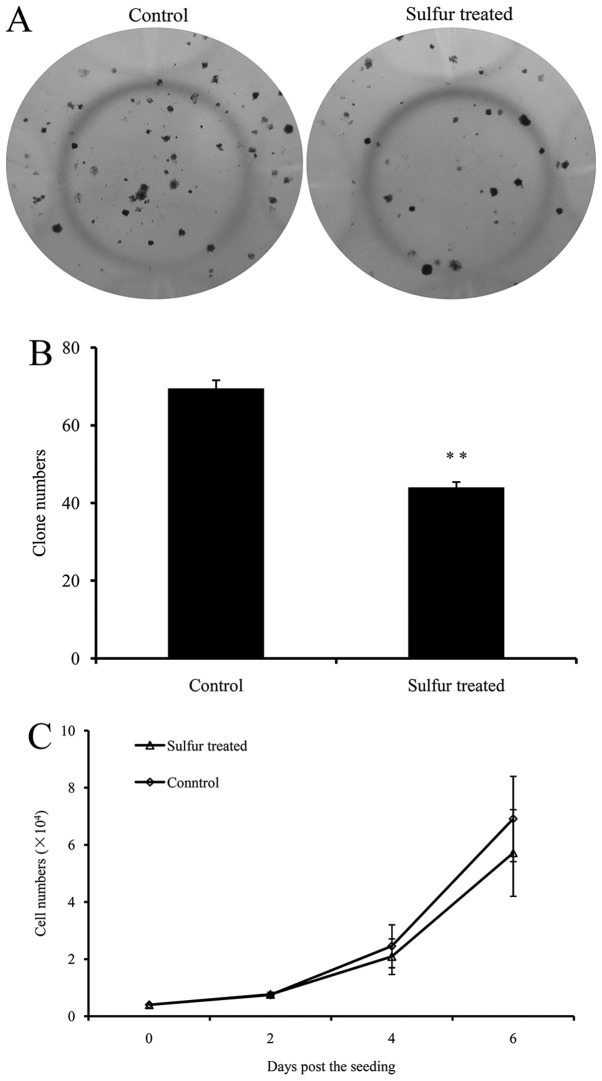
Analysis of the clonogenicity and growth rate of tumor cells. Tumor cells were separated from the 22Rv1 tumors, and cultured for analysis of the (A and B) clone-forming ability and (C) growth rate. All experiments were performed independently three times. (A) Representative experiment of clone-forming. (B) Number of clones. Clones from the three experiments was counted and averaged. ^**^P<0.01 vs. the control group. (C) Analysis of growth rate revealed no significant difference between the two groups, P>0.05.

In order to further investigate the inhibitory effects of sulfur on PCa tumor growth *in vivo*, 22Rv1 cells were separated from the xenograft tumors, and maintained in culture for at least two weeks prior to their application in the following approach. A total of 200 cells from each group were seeded in 24-well plates, respectively, and incubated to form clones (Fig. 2A). Cells from the sulfur-treated tumors formed 44.00±1.41 clones per well, which was 36.69% less than that of the control tumors (69.50±2.12 clones per well) (Fig. 2B), indicating that the clonogenicity of 22Rv1 PCa cells was significantly decreased by sulfur.

In addition, the inhibition of 22Rv1 tumor growth was maintained when sulfur was removed. The MTT method was used to measure the growth rate of 22Rv1 tumor cells; however, no significant difference was identified between cells from the two groups (Fig. 2C).

### Sulfur inhibits the expression of AR, PSA and NKX3.1 in 22Rv1 tumors

**Figure 3:**
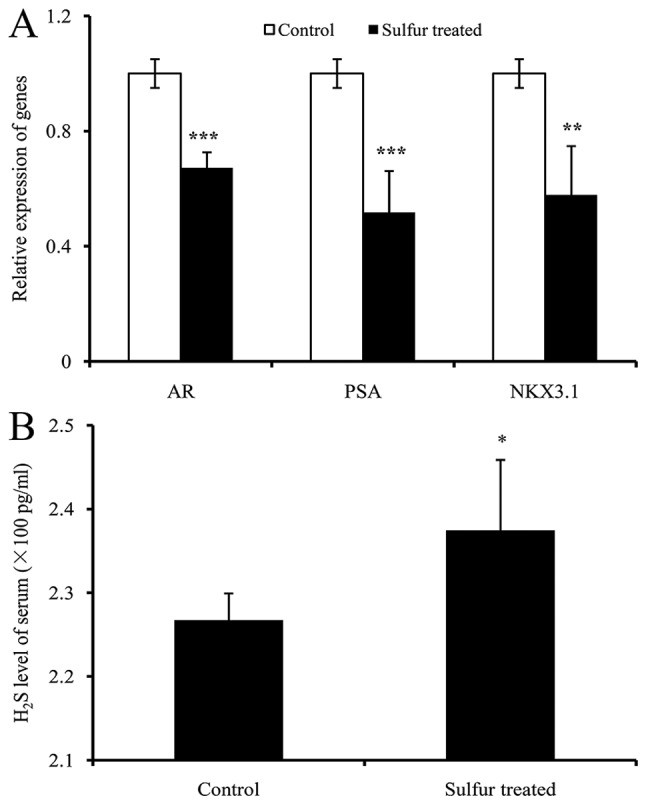
Serum H_2_S level and tumor gene expression in sulfur-treated mice. (A) Sulfur inhibited the expression of AR, PSA and NKX3.1. Total RNA was prepared from tumors and the expression of AR, PSA and NKX3.1 was measured by quantitative polymerase chain reaction. ^**^P<0.01 and ^***^P<0.001. (B) H_2_S levels in mice serum. Mouse blood was collected from the heart and serum was isolated. H_2_S levels were then measured by ELISA. ^*^P<0.05. H_2_S, hydrogen sulfide; AR, androgen receptor; PSA, prostate-specific antigen.

AR is involved in PCa progression (21), and is expressed in 22Rv1 cells, in addition to AR-regulated genes PSA (25) and NKX3.1 (26). The expression of these genes was analyzed using qPCR. As shown in Fig. 3, the expression of AR, PSA and NKX3.1 were significantly decreased in sulfur-treated tumors by 32.8, 48.2 and 42.2%, respectively (Fig. 3A). These results indicated that the downregulation of the AR signaling pathway contributes to the inhibitory effect of sulfur on PCa growth *in vivo*.

### Sulfur marginally increases H_2_S levels in mice serum

Mice serum H_2_S levels were measured by ELISA. The H_2_S level in sulfur-treated mice was marginally increased by 4.73% (237.46±8.40 pg/ml) in the sulfur-treated group compared with 226.74±3.18 pg/ml in the control group (Fig. 3B), and a significant difference was identified. The results indicated that sulfur marginally increases H_2_S levels in mice serum.

## Discussion

The present study demonstrated that sulfur significantly inhibits PCa growth in xenograft models (Fig. 1). In previous studies, inorganic sulfur has been demonstrated to inhibit the cell proliferation of breast cancer cells *in vitro* when dissolved in methanol, which is toxic to the body and difficult to use *in vivo* (27). As sulfur is not dissolved in water saline solution, it cannot be used directly for the evaluation of its anticancer effects in cell culture. In the present study, clone-forming analysis of cells separated from tumors also demonstrated the inhibitory effect of sulfur on cancer cells (Fig. 2A), and possibly contributed to its inhibitory effect observed *in vivo*. However, no significant inhibitory effect of sulfur was identified on the growth of tumor cells in the present study. The anticancer effect of sulfur may be temporary, as the growth of tumor cells was inhibited only in the environment of sulfur *in vivo*. In our study, tumors were removed from the sulfur-treated mice and the anticancer effect of sulfur was diminished. However, cells with a lower growth rate may be eliminated during long term culturing, such as in the present study.

The androgen-receptor is involved in PCa progression (21), and is an important target of numerous drugs used for PCa therapy. The present study demonstrated that sulfur significantly inhibited AR expression in 22Rv1 prostate tumors. Possibly as a result of reduced AR expression, the expression of PSA and NKX3.1, two AR-targeted genes, were also inhibited by sulfur. These results indicated that the downregulation of the AR signaling pathway was involved with the anticancer effect of sulfur. However, sulfur also markedly inhibits the growth of PCa tumors in an AR-independent way, since it also inhibited the growth of DU-145 tumors, which lacked AR expression. Novel approaches are required for understanding the AR-independent mechanism of the anticancer effect of sulfur.

As previously reported, a number of sulfocompounds inhibited the growth of various cancer cells (6–11). The anticancer capabilities were dependent (6,7) or independent (8–11) on their ability to release H_2_S. In the current study, only a marginally increased level of H_2_S was detected in the serum of sulfur-treated mice, indicating that H_2_S was not the key intermediate metabolite of sulfur. The characterization of metabolites of sulfur that exhibit anticancer activity may aid the development of anti-PCa drugs.

In conclusion, the present study revealed that sulfur significantly inhibited the growth of androgen-independent tumors. Since sulfur has been used in the clinical treatment of various diseases and is also a component of TCM, this study supports the application of sulfur in the treatment of clinical PCa, particularly recurrent PCa.

## Figures and Tables

**Table I:** Primers used for qPCR.

Gene	Primer sequences	Annealing temperature (°C)	Product length (bp)
β-actin	F: 5′-CCTGTACGCCAACACAGTGC-3′ R: 5′-ATACTCCTGCTTGCTGATCC-3′	58	211
AR	F: 5′-TTCCCTCCCTATCTAACCCTC-3′ R: 5′-TCTAAACTTCCCGTGGCATAA-3′	58	202
PSA	F: 5′-AGTCTGCGGCGGTGTTCT-3′ R: 5′-GTGGCTGACCTGAAATACCTG-3′	58	139
NKX3.1	F: 5′-AGAAAGGCACTTGGGGTCTT-3′ R: 5′-TCCGTGAGCTTAGGTTCTT-3′	60	210

F, forward; R, reverse; bp, base pairs; AR, androgen receptor; PSA, prostate specific antigen; qPCR, quantitative real-time polymerase chain reaction.
